# Duodenal GLP-1 signaling regulates hepatic glucose production through a PKC-*δ*-dependent neurocircuitry

**DOI:** 10.1038/cddis.2017.28

**Published:** 2017-02-09

**Authors:** Mengliu Yang, Jinzhi Wang, Shaobo Wu, Lei Yuan, Xiaodong Zhao, Chaohong Liu, Jing Xie, Yanjun Jia, Yerui Lai, Allan Zijian Zhao, Guenther Boden, Ling Li, Gangyi Yang

**Affiliations:** 1 Department of Endocrinology, The Second Affiliated Hospital, Chongqing Medical University, Chongqing, China; 2 Chongqing Key Lab of Child Infection and Immunity Children's Hospital of Chongqing Medical University, Chongqing, China; 3 The Key Laboratory of Laboratory Medical Diagnostics in the Ministry of Education and Department of Clinical Biochemistry, College of Laboratory Medicine, Chongqing Medical University, Chongqing, China; 4 Institute of Biomedical and Pharmaceutical Sciences, Guangdong University of Technology, Guangzhou, Guangdong, China; 5 The Division of Endocrinology/Diabetes/Metabolism and the Clinical Research Center, Temple University School of Medicine, Philadelphia, PA, USA

## Abstract

Intestinal glucagon-like peptide-1 (GLP-1) is a hormone that stimulates insulin secretion and acts as a neuropeptide to control glucose homeostasis, but little is known whether intestinal GLP-1 has any effect in the control of hepatic glucose production (HGP). Here we found that intraduodenal infusion of GLP-1 activated duodenal PKC-*δ*, lowered HGP and was accompanied by a decrease in hepatic expression of gluconeogenic enzymes and an increase in hepatic insulin signaling in rats. However, gut co-infusion of either the GLP-1 receptor antagonist Ex-9, or the PKC-*δ* inhibitor rottlerin with GLP-1, negated the ability of gut GLP-1 to lower HGP and to increase hepatic insulin signaling during clamps. The metabolic and molecular signal effects of duodenal GLP-1 were also negated by co-infusion with tetracaine, pharmacologic inhibition of *N*-methyl-d-aspartate receptors within the dorsalvagal complex, or hepatic vagotomy in rats. In summary, we identified a neural glucoregulatory function of gut GLP-1 signaling.

It is well established that nutrients can stimulate the release of gut-peptide hormones, such as cholecystokinin (CCK), pancreatic polypeptide, peptide YY and glucagon-like peptide-1 (GLP-1), which are involved in the regulation of food intake and gastrointestinal function.^1, 2, 3^ GLP-1 is a 30 amino acid peptide produced by post-translational processing of the proglucagon gene product in enteroendocrine cells (L-cells), which are most prevalent in the mucosa of the distal small intestine and colon.^4^ However, endocrine cells that produce GLP-1 also can be found throughout all regions of the porcine, rat and human small intestine.^5^ The actions of GLP-1 are mediated by specific GLP-1 receptors (GLP-1R), a G-protein-coupled receptor (GPR). In rodents and humans, a single structurally identical GLP-1R has been identified and is expressed in a wide range of tissues, including *α*-, *β*- and *δ*-cells of the pancreatic islets, lung, heart, kidney, stomach, intestine, pituitary, skin, nodose ganglion neurons of the vagus nerve, and several regions of the central nervous system (CNS) including the hypothalamus and brainstem.^5^ Central or peripheral GLP-1R activation has been shown to cause a variety of effects, including an increase in insulin release, a reduction of food intake^2, 6^ and an increase in insulin sensitivity in both rodents and humans.^7, 8, 9^ In addition, GLP-1 inhibits gastric emptying,^10^ decreases body weight^11^ and exerts a *β*-cell protective effects and beneficial cardiovascular actions.^5, 12^ Furthermore, it has also been demonstrated that the GLP-1R is expressed by intestinal mucosal cells, paneth cells and enteric neurons, that activation of receptor expression in mucosal cells is specific to GLP-1 and is regulated by neuronal inputs.^13^ Therefore, it is possible that once secreted in a paracrine fashion (local to the site of release in the small intestine), GLP-1 activates GLP-1Rs expressed in mucosal cells of the upper gastrointestinal tract through vagal afferent neurons to regulate glucose production.^14^ However, whether intestinal GLP-1 has any effect in the control of hepatic glucose production (HGP) has not been addressed.

As the majority of intestinally derived GLP-1 is rapidly degraded by the dipeptidyl peptidase-4 (DPP-4) enzyme and similar endopeptidases, the circulating GLP-1 levels are low.^15^ Currently, long-lasting active analogs or agonist of GLP-1 have already been developed as drugs.^11^ In the present study, we described a novel role of intestinal GLP-1 that triggers a gut–brain–liver neuronal network to regulate HGP by using liraglutide, a human GLP-1R agonist, and Exenatide, a natural analog of GLP-1.

## Results

### Duodenal GLP-1 lowers glucose production

**Figure 1:**
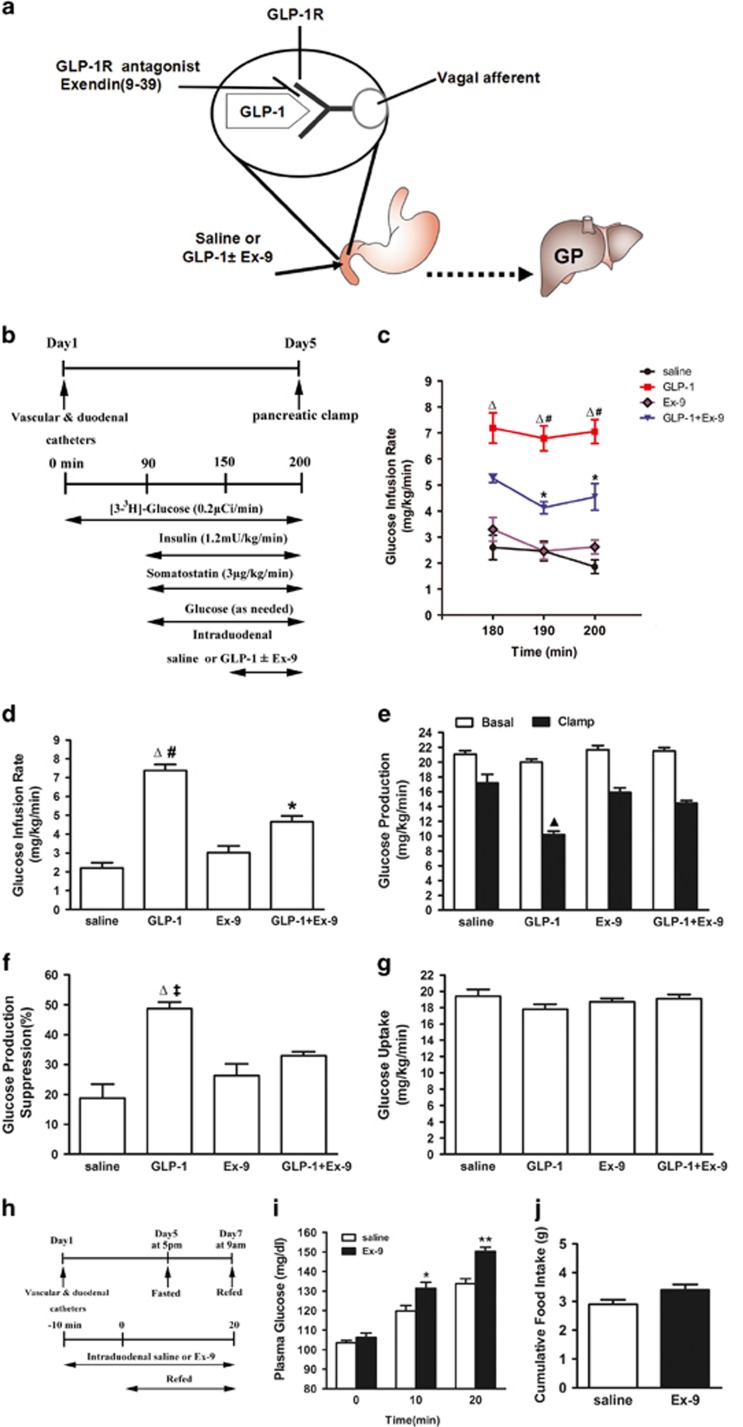
Gut GLP-1 inhibits liver glucose production through GLP-1 receptors. (**a**) Schematic representation of working hypothesis. GLP-1 with or without Ex-9 was infused through a duodenal catheter. GLP-1R, GLP-1 receptor; GP, glucose production. (**b**) Experimental procedure and clamp protocol. Duodenal catheter or venous and arterial catheters were implanted on day 1. The pancreatic (basal insulin) clamp studies were performed on day 5. (**c**) Glucose infusion rates (GIR) during the steady state of clamp (180–200 min). (**d**) Cumulative GIR during the steady state of clamp. (**e**) Hepatic glucose production (HGP). (**f**) Suppression of HGP during the clamp period expressed as the percentage reduction from basal HGP. (**g**) Glucose uptake. Data are means±S.E.M. (saline (*n*=8), GLP-1 (*n*=7), Ex-9 (*n*=6), GLP-1+Ex-9 (*n*=8)). *P<0.05, ^Δ^*P*<0.01 *versus* saline and Ex-9; ^‡^*P*<0.05, ^#^*P*<0.01 *versus* GLP-1+Ex-9; ^▴^*P*<0.01 *versus* all other groups. (**h–j**) The effect of GLP-1 receptors inhibition in the gut on glucose homeostasis during fasting–refeeding. (**h**) Schematic representation of experimental design. Duodenal catheter or venous and arterial catheters were implanted on day 1. Rats were subjected to a 40 h fasting from 1700 hours on day 5 until 0900 hours on day 7. Ten minutes before the completion of the 40 hour fast, rats were infused with intraduodenal saline or Ex-9 (*n*=6 for each group). Rats were refed on regular chow at time 0 min where food intake and blood glucose were monitored for 20 min. (**i**) Plasma glucose levels during refeeding. (**j**) Cumulative food intake during refeeding. Values are shown as mean±S.E.M. *P<0.05, **P<0.01 *versus* saline

To examine whether GLP-1R is expressed in the duodenum, GLP-1R-immunoreactivity was performed in rat duodenum tissue. GLP-1R immunoreactive cells were seen in Brunner's gland epithelium, with a predominant basolateral membrane-associated staining of epithelial cells (Supplementary Figure S1). To investigate whether intestinal GLP-1 activation regulates glucose production (Figure 1a), we selectively activated duodenal GLP-1 signaling via direct intraduodenal liraglutide or Exenatide administration *in vivo*. First, intraduodenal liraglutide or Exenatide was infused (0, 10, 30 or 60 pmol/kg/min) and demonstrated a dose-dependent increase of GIR during clamps. As shown in Supplementary Figure S2, 30 pmol/kg/min infusion of liraglutide or Exenatide increased GIR by approximately 3.4- and 3.7-fold (7.4±0.3 for liraglutide and 8.2±0.4 mg/kg/min for Exenatide *versus* 2.2±0.3 mg/kg/min for saline, all *P*<0.01). Based on considerations for preventing increase of circulating GLP-1 levels, 30 pmol/kg/min was selected for all further experiments. During the clamp, plasma glucose, insulin and free fatty acid (FFA) were maintained at basal levels (Supplementary Table S1). However, intraduodenal administration of GLP-1 increased the GIR required to maintain euglycemia, suggested an enhancement of insulin sensitivity (Figures 1c and d). This was due to an inhibition of HGP (Figure 1e) rather than changes in the rate of peripheral glucose uptake (Figure 1g). In addition, we found that the effects of Exenatide on GIR and HGP were similar to those of liraglutide (Supplementary Figure S3). Thus, Exenatide was selected for further experiments.

In another set of rats, we performed intraduodenal GLP-1 (30 pmol/kg/min) administration for 60 min and found that plasma GLP-1 levels did not change at 60 min (Supplementary Table S2). GLP-1 levels in the portal vein were slightly increased but the difference did not reach statistically significance. Therefore, an increase of GLP-1 in the duodenum lowers glucose production without changes in circulating GLP-1 or insulin levels. These results suggest that the GLP-1 doses that were used had only local (mucosa) but no systemic effects.

### Pharmacological inhibition of GLP-1 receptors disrupts the effects of duodenal GLP-1 on glucose production

To investigate whether duodenal GLP-1 infusion activates GLP-1 receptors and evaluate whether GLP-1 receptors in intestinal mucosal cells are required for the effect of gut GLP-1 on HGP, we co-infused the GLP-1 receptor inhibitor Ex-9 with GLP-1 into the duodenum (Figures 1a and b). This partially prevented GLP-1 to increase glucose infusion rates (GIRs) (Figures 1c and d) and to lower HGP (Figures 1e and f), while glucose uptake was comparable in both groups (Figure 1g). The infusion of Ex-9 alone did not affect glucose kinetics (Figures 1c–g). These results show that GLP-1 receptors are required for GLP-1 action in the gut to lower HGP.

### Duodenal GLP-1 lowers HGP through a gut–brain–liver axis

**Figure 2:**
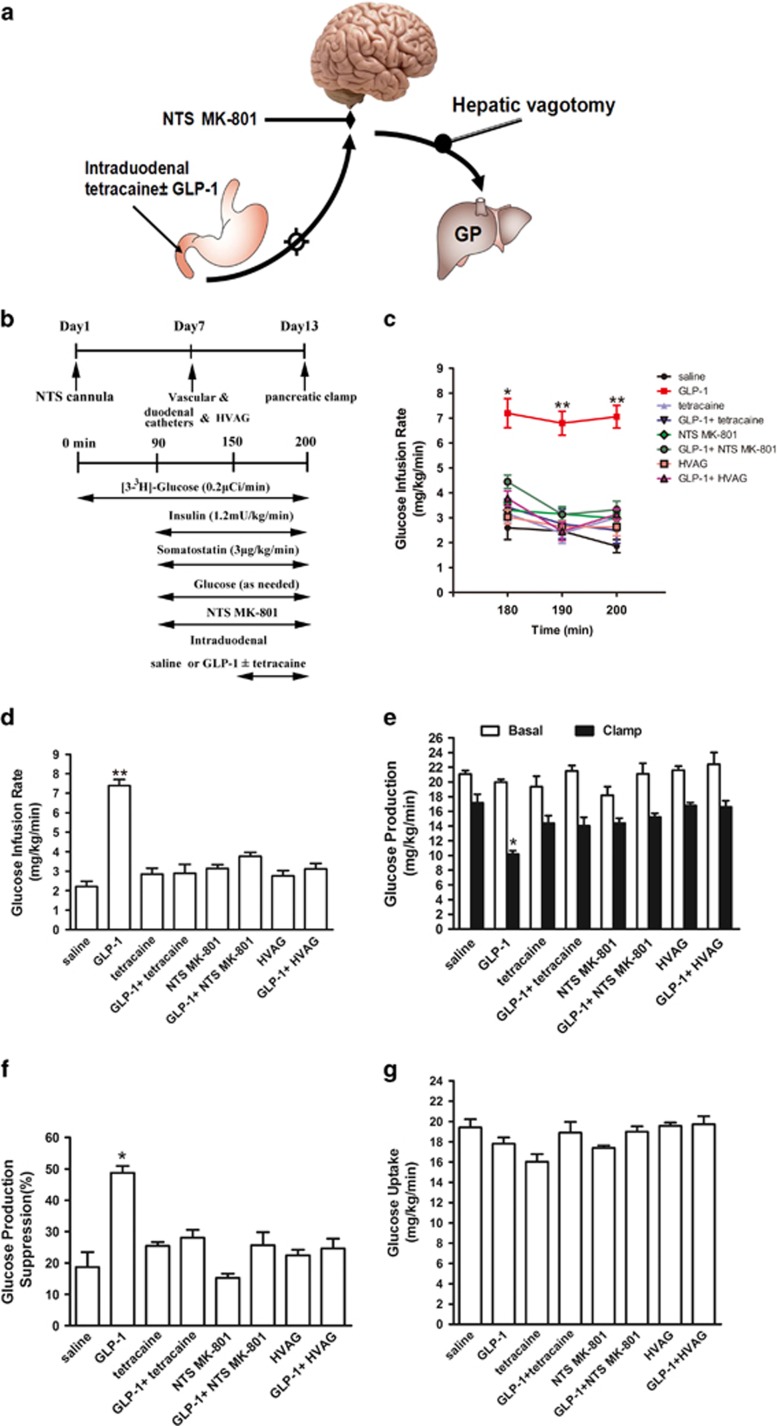
Duodenal GLP-1 inhibits glucose production by activating a gut–brain–liver neurocircuitry. (**a**) Schematic representation of the working hypothesis. Gut GLP-1 was co-infused with tetracaine through a duodenal catheter, which abolishes the ascending neuronal signal to the brain. A subgroup of rats was given MK-801, an NMDA receptor inhibitor, directly into the NTS. In another studies, gut GLP-1 was infused into rats that underwent HVAG. HVAG, hepatic vagotomy; GP, glucose production; NMDA, *N*-methyl-d-aspartate; NTS, nucleus of the solitary tract. (**b**) Experimental procedure and clamp protocol. Stereotaxic surgeries were conducted on day 1. Duodenal catheter or venous and arterial catheters were implanted on day 7. HVAG was performed immediately before the implantation of the duodenal and vascular catheters. (**c–e**) Gut GLP-1 infusion increased glucose infusion rate and lowered GP. Rats that received tetracaine in the gut, MK-801 in the NTS or HVAG failed to respond to duodenal GLP-1 to increase the glucose infusion rate and lower GP. (**f**) Suppression of GP during the clamp expressed as the percentage decrease from basal GP. (**g**) Glucose uptake was unchanged in all groups. Values are shown as mean±S.E.M. (saline (*n*=6), GLP-1 (*n*=6), Tetracaine (*n*=5), GLP-1+tetracaine (*n*=7), NTS MK-801 (*n*=5), GLP-1+NTS MK-801 (*n*=8), HVAG (*n*=6), GLP-1+HVAG (*n*=8)).*P<0.05, **P<0.01, *versus* all other groups

To examine whether gut GLP-1 lowers glucose production through a neuronal network, we co-infused the GLP-1 with the local anesthetic tetracaine, a blocker of the ryanodine receptors, into the duodenum. Intraduodenal tetracaine alone did not affect GIR and HGP (Figures 2c–g). However, when tetracaine was co-administered with GLP-1, the ability of gut GLP-1 to increase GIR (Figures 2c and d) and lower glucose production was prevented (Figures 2e and f). Glucose uptake was not affected by both duodenal GLP-1 and tetracaine (Figure 2g). Therefore, the data suggest that duodenal GLP-1 may lower HGP via activation of gut innervation of vagal afferent nerves.

Vagal afferent nerves terminate at the nucleus of the solitary tract (NTS) within the DVC. *N*-methyl-d-aspartate (NMDA) receptors in the duodenum originating neurons in the NTS of the hindbrain mediate gut signals initiated by hormone or nutrients to regulate food intake and glucose metabolism. We therefore inhibited the NMDA receptors-mediated neuronal transmission in the NTS by administration of MK-801, an NMDA receptor blocker (Figures 2a and b). MK-801 infusion alone did not affect glucose kinetics (Figures 2c–g). When NTS MK-801was co-administered with duodenal GLP-1, it negated the ability of gut GLP-1 to increase GIR (Figures 2c and d) and lower HGP (Figures 2e and f). This effect of NTS MK-801 occurred independent of changes in glucose uptake rate (Figure 2g) and insulin levels (Supplementary Table S1).

To investigate the descending pathway that mediates the effect of duodenal GLP-1 on glucose production, we next investigate whether surgical transection of the hepatic vagus nerve is sufficient to alter the effect of duodenal GLP-1 infusion on hepatic production *in vivo* (Figure 2a). We performed pancreatic-insulin clamp studies in conscious rats that underwent HVAG (Figure 2b). HAVG alone did not affect GIR and HGP. However, HAVG abolished the ability of gut GLP-1 to increase GIR (Figures 2c and d) and lower glucose production (Figures 2e and f), while glucose uptake was unchanged (Figure 2g). Collectively, these results indicate that duodenal GLP-1 increases vagal afferent neuronal activity to switch a gut–brain–liver axis to regulate HGP.

### Pharmacological inhibition of GLP-1 receptors disrupts glucose homeostasis during fasting–refeeding

Next, we investigated the effects pharmacological inhibition of gut GLP-1 receptors on glucose homeostasis during fasting–refeeding. When fasting rats are refed, the elevation of plasma glucose is restrained as a result of glucose production inhibition.^16^ We reasoned that if the nutrient-related signals can activate GLP-1 receptors to regulate HGP, the inhibition of gut GLP-1 receptors should disturb the regulation of HGP during refeeding. Rats were fasted for 40 h after 5 days of vascular and duodenal surgeries (Figure 1h). Blood glucose rose at 10 and 20 min of refeeding for the intraduodenal saline-infused rats (from 104 to 120 and then to 134 mg/dl; Figure 1i). However, after refeeding, blood glucose levels in rats infused with gut Ex-9 rose significantly higher than in gut saline-infused rats (from 106 to 131 and then to 150 mg/dl; Figure 1i), whereas food intake was similar in both groups (Figure 1j). These results suggest that GLP-1 receptors can be activated by gut-nutrient sensing to regulate glucose homeostasis and is independent of changes in food intake. The physiological relevance of the ability of the duodenal GLP-1 signaling to regulate glucose production is supported by the fact that inhibition of gut GLP-1 receptors disrupts glucose homeostasis during refeeding.

### Effect of duodenal GLP-1 receptor blockade on glucose turnover rates during duodenal lipid infusion

**Figure 3:**
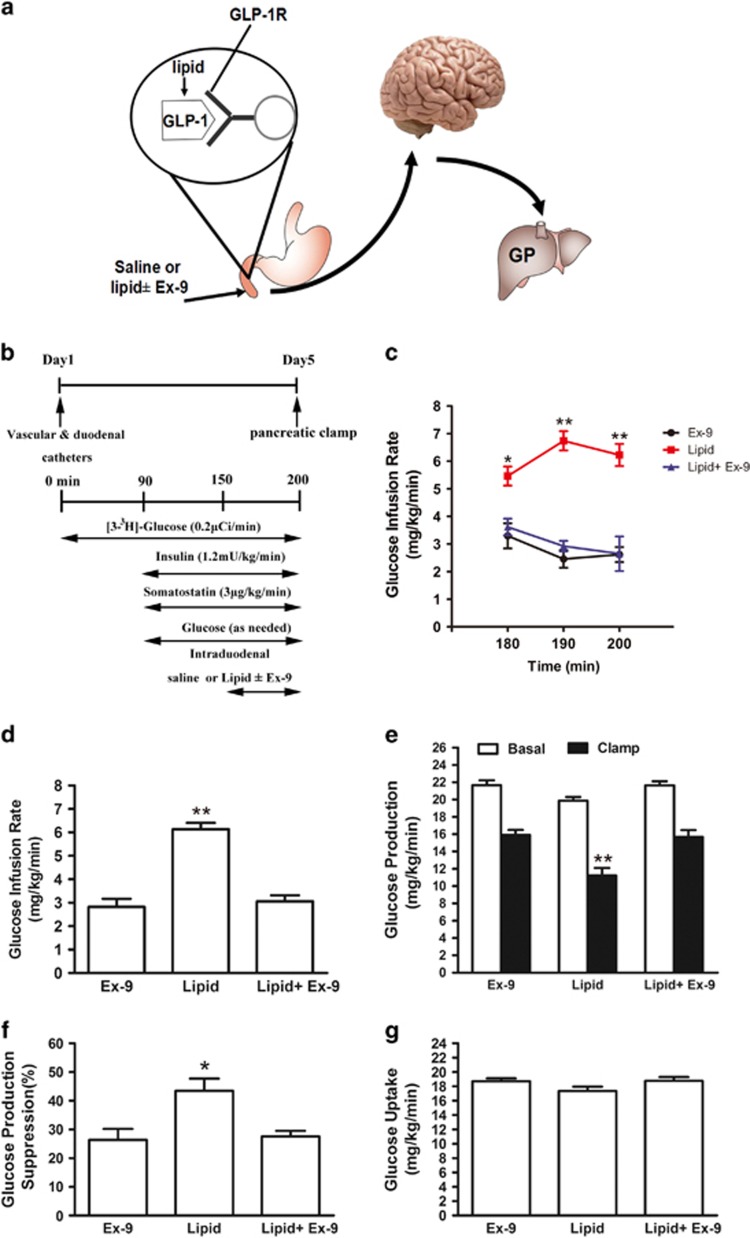
GLP-1 receptor is required for duodenal lipids to suppress hepatic glucose production. (**a**) Schematic representation of the working hypothesis. Lipid with Ex-9 or saline was infused through a duodenal catheter. (**b**) Experimental procedure and clamp protocol. (**c–f**) Gut lipids infusion increased the GIR (**c** and **d**), and decreased GP (**e** and **f**). When duodenal lipid was co-infused with Ex-9, the effects of lipids on GIR and GP were abolished. (**g**) Glucose uptake was unchanged in all groups. Values are shown as mean±S.E.M. (Ex-9 (*n*=5), lipid (*n*=8), lipid+Ex-9 (*n*=8)). *P<0.05, **P<0.01 *versus* all other groups

To evaluate whether activation of the gut GLP-1 receptor is required for nutrition sensing in the gut, we examined gut-lipid sensing in rats with and without inhibiting gut GLP-1 receptors (Figures 3a and b). Consistent with a previous report,^17^ gut-lipid infusion in rats increased GIR (Figures 3c and d) and lowered HGP during the clamps (Figures 3e and f). Notably, when the GLP-1 receptor inhibitor Ex-9 and lipid were co-infused into the duodenum, the roles of gut lipid to increase GIR (Figures 3c and d) and to lower HGP (Figures 3e and f) were reversed, whereas glucose uptake was comparable in three groups (Figure 3g). These data suggest that the activation of duodenal mucosal GLP-1 receptors is necessary for the gut-lipid-sensing mechanisms to regulate HGP. In all experimental animals, body weight did not differ between groups at the time of the clamp (Supplementary Figure S4).

### Duodenal GLP-1 activates PKC-*δ* to lower glucose production

**Figure 4:**
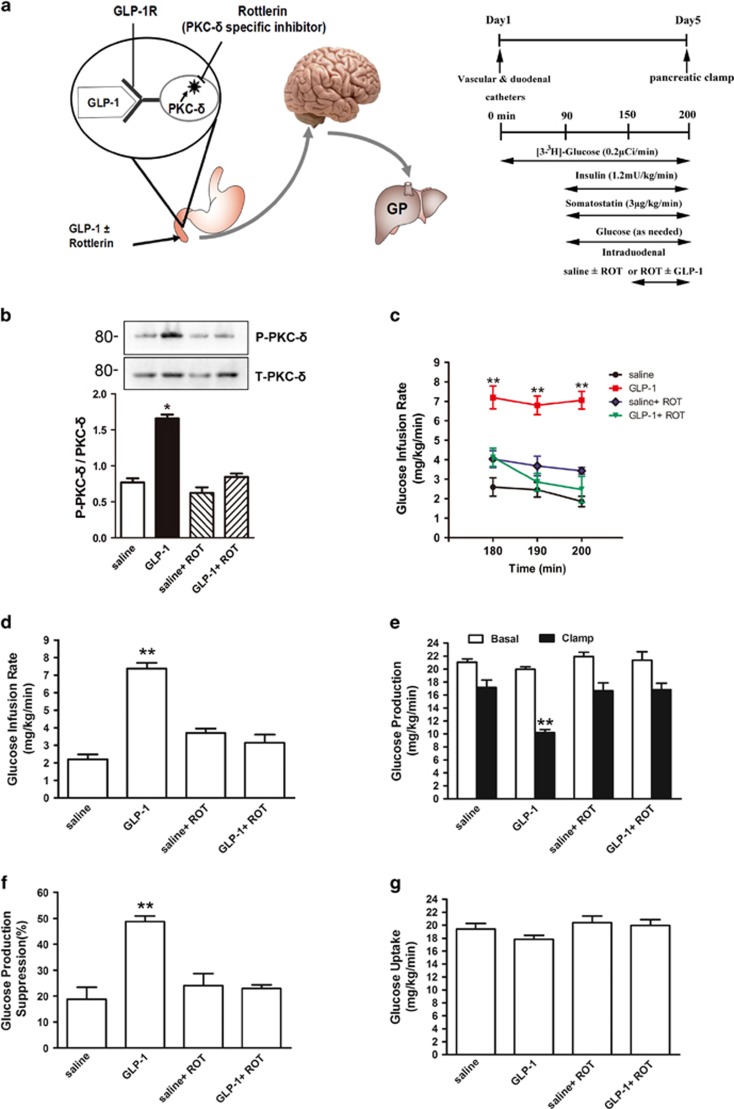
Gut GLP-1 inhibits hepatic glucose production through PKC-*δ* activation. (**a**) Schematic of working hypothesis (left), experimental procedure and clamp protocol (right). GLP-1 was infused with or without rottlerin, a PKC-*δ* specific inhibitor, through a duodenal catheter. GLP-1R, GLP-1 receptor; GP, glucose production; ROT, rottlerin. (**b**) Representative western blots (*n*=6) of PKC-*δ* in the mucosal layer of the duodenum. (**c**–**f**) Gut GLP-1 infusion increased the GIR (**c** and **d**), and decreased GP (**e** and **f**). When duodenal GLP-1 was co-infused with rottlerin, the effects of GLP-1 on GIR and GP were abolished. (**g**) Glucose uptake was comparable in all groups. Values are shown as mean±S.E.M. (saline (*n*=6), GLP-1 (*n*=6), saline+ROT (*n*=5), GLP-1+ROT (*n*=5)). *P<0.05, **P<0.01 *versus* all other groups

**Figure 5:**
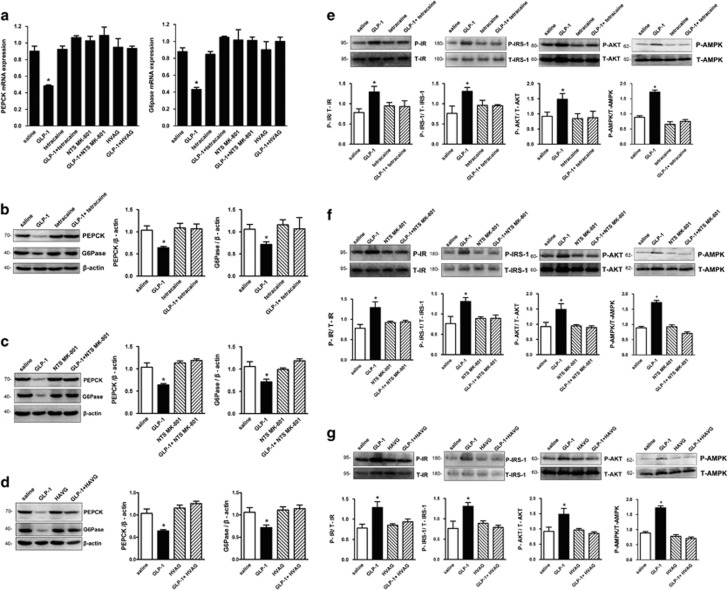
Duodenal GLP-1 suppresses hepatic PEPCK and G6Pase expression through a gut–brain–liver neurocircuitry. Intraduodenal GLP-1 infusion in rats suppressed hepatic PEPCK and G6Pase mRNA (*n*=6) (**a**) and protein expression (*n*=6) (**b–d**). In contrast, rats that received tetracaine (**b**), MK-801 in the NTS (**c**) or HVAG (**d**) failed to respond to duodenal GLP-1 to suppress hepatic PEPCK and G6Pase expression. HVAG, hepatic vagotomy; NTS, nucleus of the solitary tract. Values are shown as mean±S.E.M. (saline (*n*=6), GLP-1 (*n*=6), Tetracaine (*n*=5), GLP-1+tetracaine (*n*=8), NTS MK-801 (*n*=5), GLP-1+NTS MK-801 (*n*=7), HVAG (*n*=5), GLP-1+HVAG (*n*=7)). *P<0.01 *versus* all other groups. (**e–g**) Duodenal GLP-1 augments hepatic insulin signaling through a gut–brain–liver neurocircuitry. Representative western blots (*n*=6) and ratios of protein levels of phosphorylated InsR, IRS-1, AKT and AMPK to total InsR, IRS-1, AKT and AMPK in livers from experiments shown in Figure 2b. HVAG, hepatic vagotomy; NTS, nucleus of the solitary tract. Values are shown as mean±S.E.M. (saline (*n*=6), GLP-1 (*n*=6), Tetracaine (*n*=5), GLP-1+tetracaine (*n*=5), NTS MK-801 (*n*=5), GLP-1+NTS MK-801 (*n*=5), HVAG (*n*=5), GLP-1+HVAG (*n*=5)). *P<0.01 *versus* all other groups

It has been demonstrated that a selective activation of PKC-*δ* in the mucosal layer of the duodenum is necessary to regulate glucose metabolism.^18^ It has also been showed that brain GLP-1 signaling activates hypothalamic glucose-dependent PKC-*δ* to regulate glucose metabolism and insulin sensitivity.^19^ To explore duodenal GLP-1-dependent mechanisms, we evaluated the role of duodenal PKC-*δ* (Figure 4a). We first administered GLP-1 into the gut of rats and examined PKC-*δ* activation in the mucosal layer of the duodenum. We discovered that duodenal GLP-1 infusion significantly increased the phosphorylation of PKC-*δ* in the duodenal mucosa of rats (Figure 4b), suggesting a link between PKC-*δ* and GLP-1 signaling. To further investigate whether duodenal mucosal PKC-*δ* activation is required for duodenal GLP-1 signaling to regulate glucose metabolism, we inhibited duodenal mucosal PKC-*δ* via intraduodenal co-infusion of GLP-1 with the PKC-*δ* inhibitor rottlerin (Figure 4a). This fully abolished the ability of GLP-1 to increase GIR (Figures 5c and d) and lower HGP (Figures 4e and f), whereas gut rottlerin infusion alone had no effects (Figures 4c–g) and glucose uptake was comparable in all groups (Figure 4g).

### Effect of duodenal GLP-1 on hepatic expression of PEPCK and G6Pase

Because duodenal GLP-1 significantly inhibited HGP, we examined whether expressions of PEPCK and G6Pase, two key gluconeogenic enzymes, were altered by duodenal GLP-1. Rats that received duodenal GLP-1 had significant decreased hepatic PEPCK and G6Pase mRNAs and protein expression compared with the duodenal saline control rats (Figures 5a and b). These results indicate that duodenal GLP-1 strengthened the inhibitory effects of insulin on PEPCK and G6Pase in the liver and led to decreased HGP. However, co-infusion of intraduodenal tetracaine with GLP-1 fully negated the ability of GLP-1 to inhibit hepatic expression of PEPCK and G6Pase mRNAs and proteins, whereas gut tetracaine alone had no effects (Figures 5a and b). Similar to the effect of intraduodenal tetracaine, co-infusion of NTS MK-801 with intraduodenal GLP-1 also prevented the ability of intraduodenal GLP-1 to decrease the expression of PEPCK and G6Pase mRNAs and proteins in the liver (Figures 5a and c). In addition, in HVAG rats, intraduodenal GLP-1 infusions did not alter the expression of PEPCK and G6Pase mRNA and protein in the liver (Figures 5a and d). Hepatic G6Pase and PEPCK expression was comparable in gut saline with HVAG and gut saline alone groups (Figures 5a and d). Therefore, inactivation of the hepatic branch of the vagus nerve was sufficient to disrupt hepatic autoregulation of PEPCK and G6Pase expression during intraduodenal GLP-1 infusions.

### Effects of intraduodenal GLP-1 on insulin signaling in liver

To determine the mechanisms by which duodenal GLP-1 inhibited HGP, we examined hepatic phosphorylation levels of some insulin signaling molecules by western blots. As shown in Figure 5e, upon duodenal GLP-1 infusion, the phosphorylation of InsR, IRS-1, AKT and AMPK were significantly increased. When tetracaine was concomitantly infused with GLP-1 into the duodenum, the gut GLP-1 mediated increase in hepatic InsR, IRS-1, AKT and AMPK phosphorylation was completely prevented (Figure 5e). To test whether NTS NMDA receptors are required for the gut GLP-1-induced enhancement of hepatic insulin signals, we directly infused the NMDA receptor inhibitor MK-801 into the NTS. This alone did not affect hepatic insulin signals (Figure 5f). Importantly, coadministration of intraduodenal GLP-1 with NTS MK-801 prevented gut GLP-1 to enhance InsR, IRS-1, AKT and AMPK phosphorylation in the liver (Figure 5f). Furthermore, to evaluate whether NTS NMDA receptors relay the signal generated by gut GLP-1 to the liver and enhance the hepatic insulin signaling cascade, we repeated duodenal GLP-1 infusion experiments in rats that underwent the hepatic vagal branch vagotomy. When duodenal GLP-1 infusion was co-administered with HVAG, the ability of gut GLP-1 to enhance hepatic InsR, IRS-1, AKT and AMPK phosphorylation was abolished, whereas HVAG alone did not affect hepatic insulin signals (Figure 5g). Collectively these data indicate that the NTS relays a signal generated by intestinal GLP-1 to the liver to enhance hepatic insulin signaling. Finally, to investigate the effects of gut GLP-1 on insulin signaling molecules in other insulin target issues, we assessed the phosphorylation levels of InsR and Akt in adipose and muscle tissues during duodenal GLP-1 infusion. We found that neither gut GLP-1 nor any other administration affected the phosphorylation of insulin signaling molecules in both adipose and muscle tissues (Supplementary Figures S5A and B).

## Discussion

Diabetes and obesity are characterized by excessive rates of HGP.^20^ Therefore, it appears important to assess whether different gut-peptide hormones, such as CCK, peptide YY and GLP-1(via intraduodenal infusion or oral ingestion) regulate glucose production.

In recent years, a series of papers published by the Lam laboratory^17, 18, 21, 22^ has highlighted the existence of a gut–brain–liver axis in the regulation of glucose homeostasis through combination with duodenal tetracaine infusion (inhibiting gut vagal afferent fibers),^23^ MK-801 infusion (the NMDA receptor inhibitor)^24^ and hepatic branch vagotomy.^25^ Using this neural network, Lam and co-workers^17, 18^ and other groups^26^ investigated the effects of intestinal nutrients and hormones on HGP and fat metabolism. Along this line of evidence, it is important to continue dissecting the physiological role of some novel signaling molecules within the duodenum in regulating glucose homeostasis via this gut–brain–liver axis. Therefore, the main objective of this study was to evaluate for the first time the role of the gut GLP-1 to regulate HGP and hepatic insulin signal. In this article, we have specifically shown the following: (1) the gut GLP-1 participates the regulation of HGP and hepatic insulin signaling in rodents; (2) gut GLP-1 requires activation of GLP-1R in the intestinal mucosal layer; (3) activation of PKC-*δ* in the gut mucosal layer is required for gut GLP-1 to lower HGP, suggesting that PKC-*δ* activation lies downstream of GLP-1 signaling; (4) a neuronal network activation is required for gut GLP-1 to lower glucose production in normal rats. These data highlight a previously unappreciated role of duodenal GLP-1 signaling in the neural regulation of glucose homeostasis.

In previous studies, the main hormonal effect of GLP-1 was considered to be stimulation of glucose-induced insulin secretion.^27^ In this study, we postulate that GLP-1 released from the duodenal mucosa acts at local GLP-1 receptors in vagal afferent nerve that innervate the lamina propria of the mucosa. Therefore, to examine whether intestinal GLP-1 regulates glucose production, we activated duodenal GLP-1 signaling via direct intraduodenal GLP-1 administration. We found that an increase of GLP-1 in the duodenum lowers glucose production, inhibits the hepatic expression of G6Pase and PEPCK (two key gluconeogenic enzymes) and enhances hepatic insulin signaling. These changes were independent of changes in circulating GLP-1 or insulin levels. These findings suggested that the increase of gut GLP-1 triggers a gut hormone sensor to inhibit HGP and favor hepatic glycogen deposition. Furthermore, because co-infusion of the GLP-1 receptor inhibitor Ex-9 with GLP-1 into the duodenum abolished the ability of GLP-1 to inhibit HGP, duodenal GLP-1 receptors appear to be required for this potent control of gut GLP-1 on HGP.

Given that intestinal nutrients, such as lipids or glucose, stimulate local release of gut-peptide hormones. We investigated whether GLP-1 receptors in the gut are required for the gut-nutrient regulation on HGP with fasting–refeeding studies and intraduodenal coadministration of intralipid with Ex-9. We found that gut Ex-9 administration disrupted glucose homeostasis during refeeding and reversed the ability of gut lipids to increase GIR and lower HGP during the pancreatic-euglycemic clamps (PECs). These data strengthen the role of intestinal GLP-1 on glucose production and further indicate that GLP-1 receptors are required for gut-nutrient sensing. Noteworthy, the previous studies from Cheung *et al.*^17^ demonstrated that CCK-A receptors are also required for gut-nutrient sensing. It is also of interest that Beglinger *et al.*^27^ found that blockade of CCK-1 receptors by a specific antagonist (DEXLOX) markedly reduced duodenal lipid stimulated GLP-1 release. They and others^28^ proposed that the specific products of fat digestion, including long-chain fatty acids, stimulate the release of CCK; CCK in turn acts on CCK-1 receptors, which then initiate a series of digestive actions including modulation of GLP-1 release (a fat–CCK–GLP-1 axis). Therefore, it is likely that both CCK and GLP-1 are required for gut-nutrient sensing to lower glucose production.

Recently, it has been reported that PKC-*δ* is expressed in the duodenum may be involved in the regulation of glucose production.^29^ In this study, we demonstrate that activation of the duodenal PKC-*δ* is necessary for duodenal GLP-1 to regulate glucose metabolism, thereby revealing a duodenal GLP-1 and PKC-*δ* signaling axis that lowers HGP in the presence of basal insulin levels. This finding also suggests that activation of duodenal PKC-*δ* may lie downstream of GLP-1 signaling. However, an important unanswered question remains as to how duodenal GLP-1- triggers a neuronal network leading to the inhibition of glucose production. We propose that gut GLP-1-PKC-*δ* signaling first elicits an afferent neuronal signal to the hindbrain. The hindbrain then relays this signal to the liver to lower HGP. Here we used three independent yet complementary approaches to investigate whether a neuronal network involving the gut, brain and liver mediates the suppressive effects of duodenal GLP-1 on HGP.

We first infused the topical anesthetic tetracaine to inhibit the neurotransmission of vagal afferent fibers innervating the upper intestine in the absence or presence of gut GLP-1. Tetracaine administration significantly reduced the ability of duodenal GLP-1 to enhance hepatic insulin signaling, to inhibit gluconeogenic enzymes and to lower HGP, confirming that gut GLP-1 triggers a neuronal signal in the intestine to lower HGP.

The vagal afferent nerves terminate at the nucleus of the NTS within the DVC. Glutamatergic neurotransmission has been demonstrated in gut-recipient NTS neurons, and NMDA receptors have been localized to vagal afferent terminals in the hindbrain NTS.^30, 31^ NMDA receptors in the gut-recipient neurons in NTS of the hindbrain mediate gut signals initiated by hormones or nutrients to regulate food intake and glucose homeostasis.^2, 3^ We inhibited NMDA receptor-mediated neuronal transmission in the DVC via direct NTS-targeted administration of the NMDA receptor blocker MK-801. As expected, NTS MK-801 fully reversed the ability of gut GLP-1 to enhance hepatic insulin signaling and lower glucose production. These results support the idea that NTS transmission relays afferent neuronal signals triggered by gut GLP-1 to lower HGP and enhance hepatic insulin signaling.

The parasympathetic vagus nerve appears to be a crucial link in the CNS control of hepatic metabolism.^32^ It has been reported that leptin^33^ and GLP-1^34^ directly control liver and/or WAT metabolism, and most of those signals require an intact sympathetic nervous system. To evaluate whether the hindbrain relays signals induced by gut GLP-1 to the liver to lower HGP, we repeated the gut GLP-1 infusion protocol in rats that underwent vagotomy of the hepatic branch to block the neurocommunication between the brain and liver. As expected, hepatic vagotomy inhibited the ability of gut GLP-1 to increase GIR, lower HGP and enhance hepatic insulin signaling. These results strengthen our hypothesis that the upper-gut-GLP-1 induced neuronal signals are first processed by the NTS and are then relayed to the liver. Collectively, it appears plausible that upper intestine nutrients, such as lipids, stimulate the release of GLP-1 from mucosal cells which then bind to GLP-1 receptors on vagal afferents nerves in the duodenum and activate mucosal PKC-*δ*. By activating vagus afferent nerves, hormone or nutrient derived signals arrive at the NTS and activate the neurons in the hindbrain region and NMDA receptors. Finally, these signals are relayed from the NTS to the liver via the efferent branch of the hepatic vagal nerve to lower HGP (Supplementary Figure S6).

In conclusion, our data show that a rise in intestinal GLP-1 activates GLP-1 receptors and stimulates duodenal mucosal PKC-*δ* activation triggering a gut–brain–liver neuronal axis which lowers glucose production in normal rodents.

## Materials and Methods

### Animal preparation, stereotaxic surgery, duodenal and intravenous cannulations, and selective hepatic branch vagotomy

These sections are described in detail in Supplementary Information.

### Intraduodenal infusions and treatments

To examine the dose-dependent effects of intraduodenal GLP-1 on the GIR during PECs, different concentrations of liraglutide (Novo Nordisk, Copenhagen, Denmark) or Exenatide (AstraZeneca, Cambridge, UK) (0, 10, 30, 60 pmol/l) were continuously infused to the duodenum through the duodenal catheter during the 150–200 min of clamp (Supplementary Figure S2). For all experiments, we infused GLP-1 (Exenatide) into the duodenum at 30 pmol/kg/min, which was shown to suppress HGP (Figure 1). In addition, the following materials were also infused into the duodenum at a rate of 0.6 ml/h during the pancreatic clamp when required: (1) saline; (2) GLP-1 receptor antagonist Exendin Fragment 9–39 (Ex-9) (Aladdin, Shanghai, China; 300 pmol/kg/min);^35^ (3) tetracaine (Sigma-Aldrich, Shanghai, China; 0.01 mg/min); (4) 20% intralipids (Sino-Swed Pharmaceutical Corp. Ltd, Beijing, China; 0.03 kcal/min) and (5) rottlerin: a PKC-*δ* inhibitor (Sigma-Aldrich; 60 *μ*mol/l).^29^

### PEC procedure

Rats were limited to 20 g food to ensure the same nutritional post-absorptive status at the night before the PECs. A continuous infusion of high-performance liquid chromatography-purified [3-H^3^] glucose (PerkinElmer, Waltham, MA, USA; 6 *μ*Ci bolus, 0.2 *μ*Ci/min) was initiated at 0 min and maintained throughout the experiment to assess glucose metabolism. The PECs was started 90 min after the tracer infusion to allow the tracer to reach steady state. Insulin (1.2 mU/kg/min) and somatostatin (3 *μ*g/kg/min) were continuously infused, and a variable infusion of 25% glucose was started and adjusted every 10 min to maintain blood glucose at ~7 mmol (Figure 1b). Blood glucose was monitored with a portable glucometer and various study solutions were infused through the right internal jugular vein catheter. Intraduodenal infusions were initiated from *t*=150 to 200 min to determine the effects of different gut treatments on glucose production. In a subgroup of rats undergoing NTS treatment procedures, MK-801, an NMDA receptor inhibitor (Sigma-Aldrich, 0.03 ng/min), was initiated at *t*=90 to 200 min until the end of the clamp. The 100 *μ*l of blood samples were obtained from the left carotid artery catheter at 0, 60, 90, 180, 190 and 200 min for determination of insulin, GLP-1, FFA, triglyceride, total cholesterol and glucose-specific activity. Each blood sample was replaced by the same volume of fresh whole blood from a donor rat. At the end of the clamp, the rats were anesthetized, portal vein blood was obtained and tissue samples were freeze-clamped *in situ* with aluminum tongs precooled in liquid nitrogen and stored at −80 °C for further analysis.

### Fasting–feeding study

After the vascular and duodenal surgery, rats were allowed 5 days to recovery. A subgroup of rats was fasted beginning at 5 PM the day before the onset of the experiment. Rats were refed after fasting for 40 h. Rats were subjected to a continuous intraduodenal infusion of either saline or Ex-9 (300 pmol/kg/min) for 30 min (Figure 1h). Blood glucose levels were measured at *t*=0, 10 and 20 min. Cumulative food intake was measured at the end of this protocol.

### Immunohistochemistry, RNA extraction and quantitative real-time RT-PCR, western blot analyses and analytical procedure

These sections are described in detail in Supplementary Information.

### Statistical analyses

All groups from our data showed normal variance, and thus we analyzed results using an unpaired Student's *t*-test for the analyses of two groups or ANOVA followed by Tukey's *post hoc* test when comparisons were made for more than two groups. All results are presented as means±S.E.M. *P*<0.05 was considered significant. Data between *t*=60–90 min and *t*=180–200 min were averaged for the basal and clamp conditions, respectively. No blinding was done for any of the experimental procedures.
