# Supplementary material for: Duodenal GLP-1 signaling regulates hepatic glucose production through a PKC-δ-dependent neurocircuitry
Source: Cell Death Dis. 2017 Feb 9;8(2):e2609–. doi: 10.1038/cddis.2017.28 (PMC5386475; doi:10.1038/cddis.2017.28)
Supplement: Supplementary Information [file cddis201728x1.doc]

**Supplementary information**

**Supplementary methods**

**Animal preparation**

A total of 210 9-week-old Male SD rats (300-350g) were housed in individual cages and maintained on a standard light-dark cycle. The rats had access to chow and water ad libitum and were given 7 days to acclimatize before the designated surgeries were performed. All the animal study protocols were reviewed and approved by the Animal Experimentation Ethics Committee of Chongqing Medical University. Except as noted below, no animals were excluded from analysis, and rats were randomly assigned into surgical groups detailed below with no blinding.

**Stereotaxic surgery**

Bilateral catheters (PlasticsOne, Roanoke, VA) were inserted into the dorsal vagal complex (DVC) targeting the nucleus of the solitary tract (NTS) into a subgroup of rats. The stereotaxic brain surgeries were performed 7 days before the duodenal and intravenous cannulations, allowing sufficient recovery time. The coordinates were 0.0 mm on the occipital crest, 0.8 mm (left and right) lateral to the midline, and 7.9 mm below the skull surface. Proper placement of the cannulas was confirmed histologically in brain slices prepared postmortem (Figure S7) as described^1^.

**Duodenal and intravenous cannulations**

A catheter was inserted into the proximal duodenum (approximately 1.5-2 cm downstream of the pyloric sphincter). We also placed indwelling catheters into the internal jugular vein and carotid artery for infusion and blood sampling. Recovery from surgery was detected by measuring food intake and weight for 3 to 4 days after surgery. These surgical procedures were performed as described^2, 3^.

**Selective hepatic branch vagotomy**

Separate cohorts of rats were subjected to hepatic branch vagotomy (HVAG). Briefly, an incision was made into the ventral midline. The hepatic branch of the ventral subdiaphragmatic vagal trunk was identified and transected by microcautery, severing the hepatic vagus. Anatomical nerve transactions were verified as described^4^.

**Immunohistochemistry for GLP-1R protein expression in** [**duodenum**](javascript:void(0);)

[Duodenum](javascript:void(0);) sections were preincubated in 0.5% Triton (Sigma Aldrich) and 1.5% bovine serum albumin (BSA) (Sigma Aldrich) in PBS for 15 min at room temperature. Successively, the sections were incubated with GLP-1R polyclonal antibody (Bioss, Beijing, China, bs-1559R) at the ﬁnal dilution of 1 : 100 in 0.5% Triton and 1.5% BSA in PBS for 24 h at 4 ºC. The immunoreaction was revealed by using the horseradish peroxidase–labeled sheep anti-rabbit antibody 1 : 500 for 1 h at 37 ºC.

**RNA Extraction and Quantitative Real-Time RT-PCR**

Total RNA was isolated from frozen tissue with TRIzol reagent (TakaraBio, Otsu, Japan) according to the manufacturer’s instructions. Quantitative real-time RT-PCR (qRT-PCR) was performed with a SYBR Green PCR kit (Takara Bio, Otsu, Japan) and a Corbett Rotor-Gene 6000 real time PCR system (Bio-Rad, California, USA). mRNA expressions were analyzed with the comparative threshold cycle method and normalized with β-actin. The following primers were used: 5’-CCCTGAACCCTAAGGCCAACCGTGAAAA-3’and 5’-TCTCCGG AGTCCATCACAA TGCCTGTG-3’ for β-actin, 5’-CACCTTGACACTACACCCTT-3’ and 5’-GTGGCTGTGAACACCTCT-3’ for glucose-6-phosphatase (G6Pase), and 5’-AGTCA CCATCACTTCCTGGAAGA-3’ and 5’-GGTGCAGAATCGCGAGTT-3’ for phosphoenolpyruvate carboxykinase (PEPCK).

**Western blot analyses**

Tissue samples were homogenized, and protein levels were measured with a BCA quantification kit (Beyotime Biotechnology, Shanghai, China). Protein lysates were subjected to 8% SDS-PAGE and transferred to polyvinylidene difluoride membranes. Membranes were probed at 4°C in the presence of 1:1,000 dilutions of primary antibodies including against the insulin receptor (InsR) (#3025) /phospho-InsR (#3024), insulin receptor substrate 1 (IRS-1) (#2382)/phospho-IRS-1 (#2386), AMP-dependent protein kinase (AMPK) (#2532)/phosphor-AMPK (#2531), protein kinase C (PKC)-δ (#2056)/phospho-PKC-δ (#2055), AKT kinase (AKT) (#9272)/ phospho- AKT (#4060) (Cell Signaling Technology, Beverly, MA, USA); G6Pase(Abcam, Cambridgeshire, UK, ab83690) and PEPCK (Santa Cruz Biotechnology, Dallas, TX, USA, sc-74823); and β-actin (Santa Cruz Biotechnology, Dallas, TX, USA, sc-47778). After being washed three times with Tris-buffered saline, the membranes were incubated with horseradish peroxidase–labeled sheep anti-rabbit antibody for 1 h. Finally, the blots were visualized with enhanced chemiluminescence. Band intensity was quantified with the Cool Imager^TM^ (Viagene Biotech Inc, Florida, USA), and quantification was performed with Quantity One analysis software (Bio-Rad).

**Analytical procedure**

Blood glucose was examined with the glucose oxidase method, plasma insulin was measured with a commercial insulin radioimmunoassay kit (HTA CO. LTD., Beijing, China). FFA was determined spectrophotometrically with an anacyl-CoA oxidase-based colorimetric kit (Wako Pure Chemical Industries, Osaka, Japan). TG and TC levels were measured with enzymatic colorimetric kits. Circulating and portal vein GLP-1 levels were measured using a commercial enzyme-linked immunosorbent assay kit (CUSABIO, Wuhan, China). Plasma [3-H^3^] glucose–specific activity was measured as described ^5^.

**Supplementary references**

1. Treece BR, Covasa M, Ritter RC, Burns GA. Delay in meal termination follows blockade of N-methyl-D-aspartate receptors in the dorsal hindbrain. Brain Res 1998; **810**(1-2)**:** 34-40.

2. Kokorovic A, Cheung GW, Breen DM, Chari M, Lam CK, Lam TK. Duodenal mucosal protein kinase C-delta regulates glucose production in rats. Gastroenterology 2011; **141**(5)**:** 1720-7.

3. Wang PY, Caspi L, Lam CK, Chari M, Li X, Light PE et al. Upper intestinal lipids trigger a gut-brain-liver axis to regulate glucose production. Nature 2008; **452**(7190)**:** 1012-6.

4. la Fleur SE, Ji H, Manalo SL, Friedman MI, Dallman MF. The hepatic vagus mediates fat-induced inhibition of diabetic hyperphagia. Diabetes 2003; **52**(9)**:** 2321-30.

5. Li X, Yang M, Wang H, Jia Y, Yan P, Boden G et al. Overexpression of JAZF1 protected ApoE-deficient mice from atherosclerosis by inhibiting hepatic cholesterol synthesis via CREB-dependent mechanisms. Int J Cardiol 2014; **177**(1)**:** 100-10.

**Supplementary Figures**


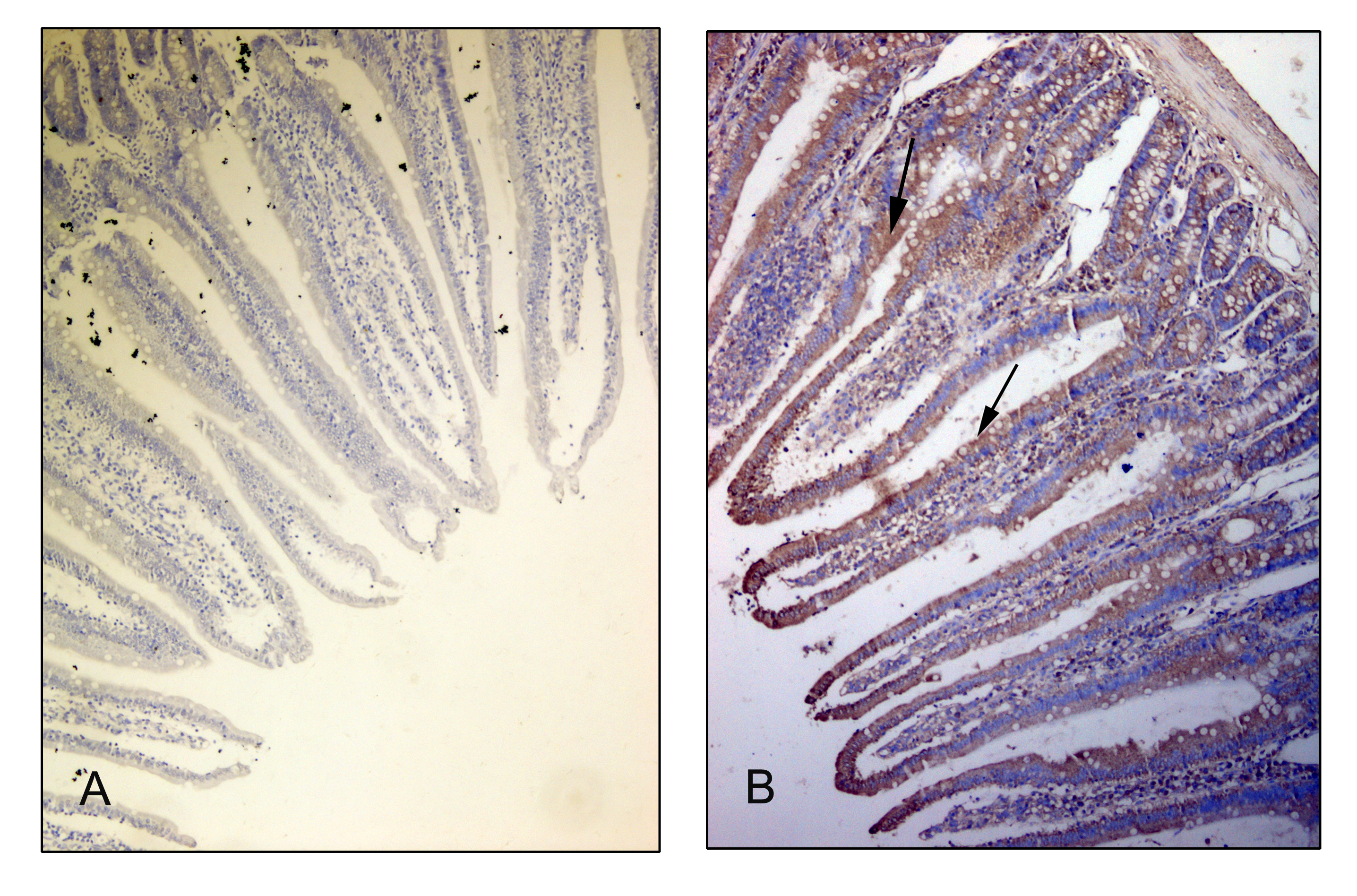
\

**Figure S1.** **Identification of GLP-1R immunoreactive cells in the rat** [**duodenum**](javascript:void(0);)**.** [Duodenum](javascript:void(0);) sections were immunostained for GLP-1R with saline **(A)** or GLP-1R polyclonal antibody **(B)**. Arrows highlight that the GLP-1R immunopositive area. Original magnification, ×100.


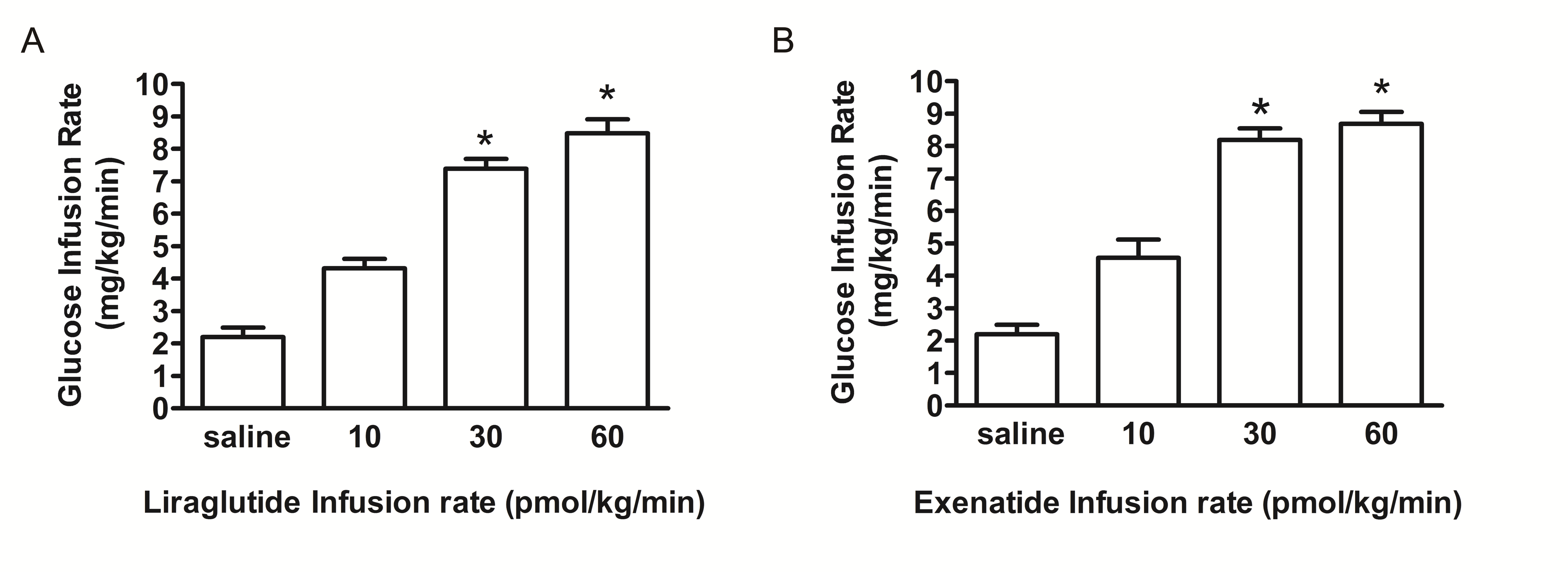


**Figure S2.** **Gut GLP-1 infusion increased glucose infusion rate in dose dependent manner during the steady-state of clamp. (A)** Liraglutide. **(B)** Exenatide. Values are shown as mean ± SEM. n =5 rats/group. **P*< 0.01 *vs*. saline group.


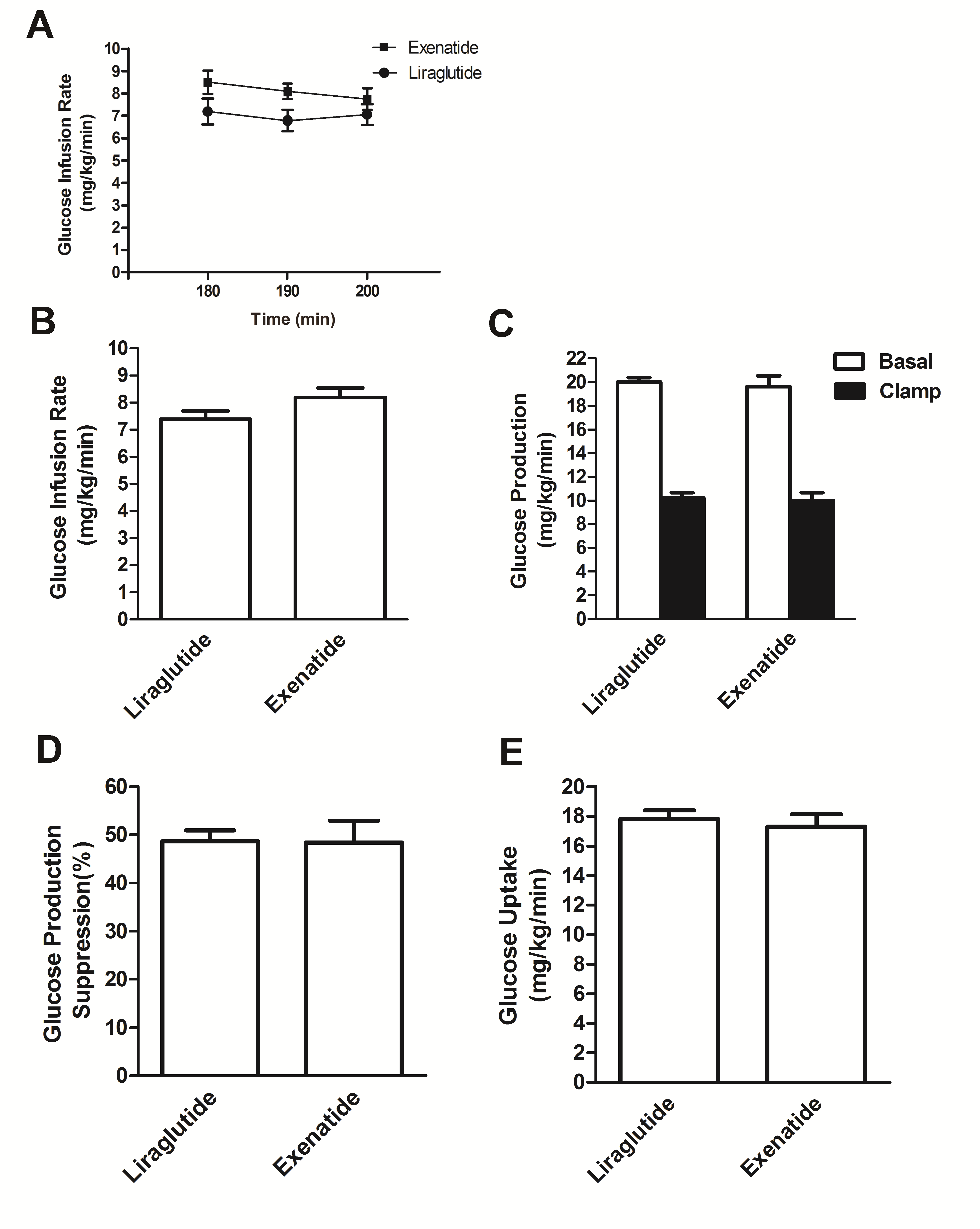


**Figure S3.** **The effects of duodenal liraglutide and Exenatide infusion on the GIR and HGP during clamp. (A)** Glucose infusion rates (GIR) during the steady-state of clamp (180-200 min). **(B)** Cumulative GIR during the steady-state of clamp. **(C)** Hepatic glucose production (HGP). **(D)** Suppression of HGP during the clamp period expressed as the percentage reduction from basal steady state HGP. **(E)** Glucose uptake. Data are means ± SEM (n= 5 rats/group).

**
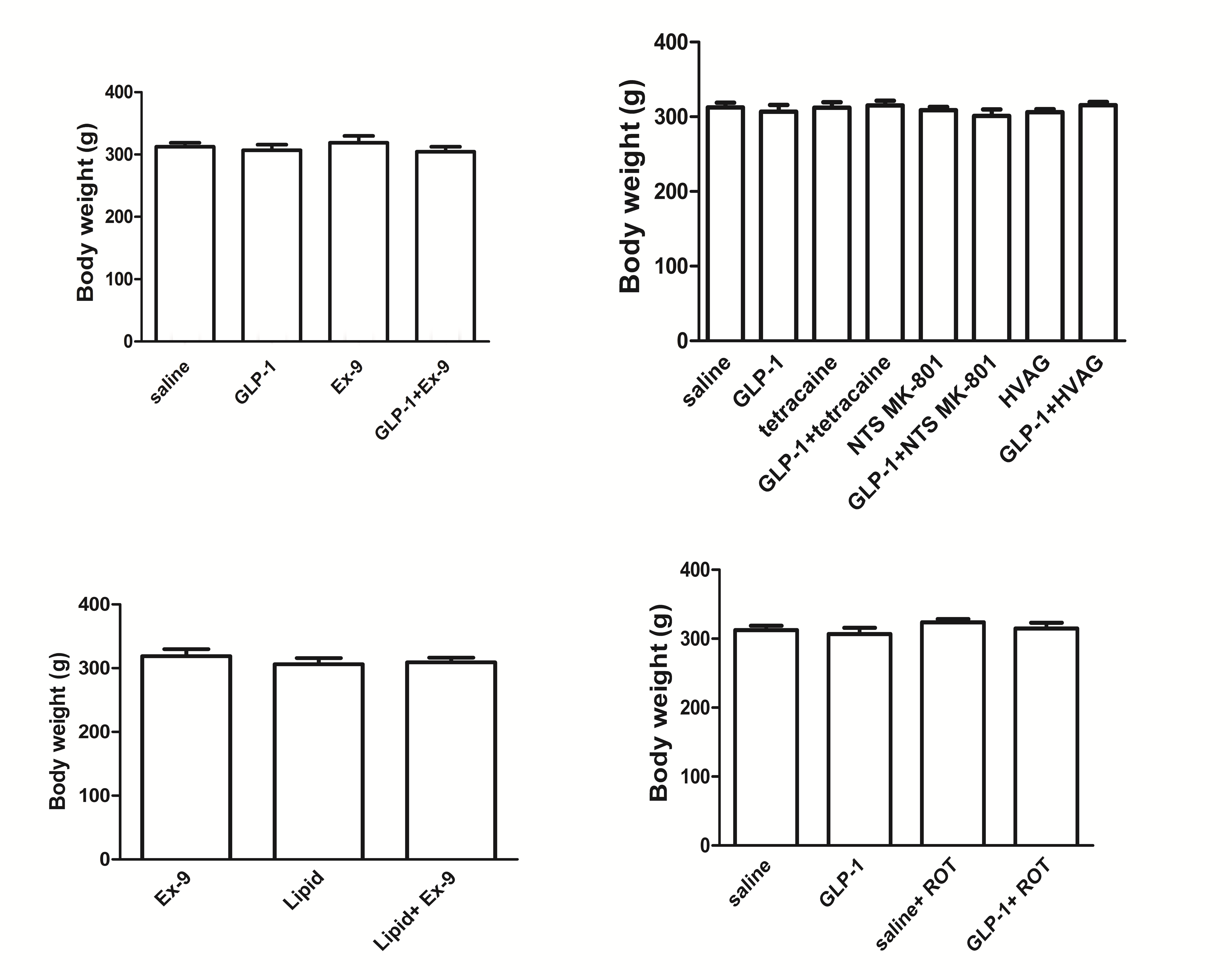
**

**Figure S4. The body weight for each experimental group at the time of the clamp.**

Data are means ± SEM.

**
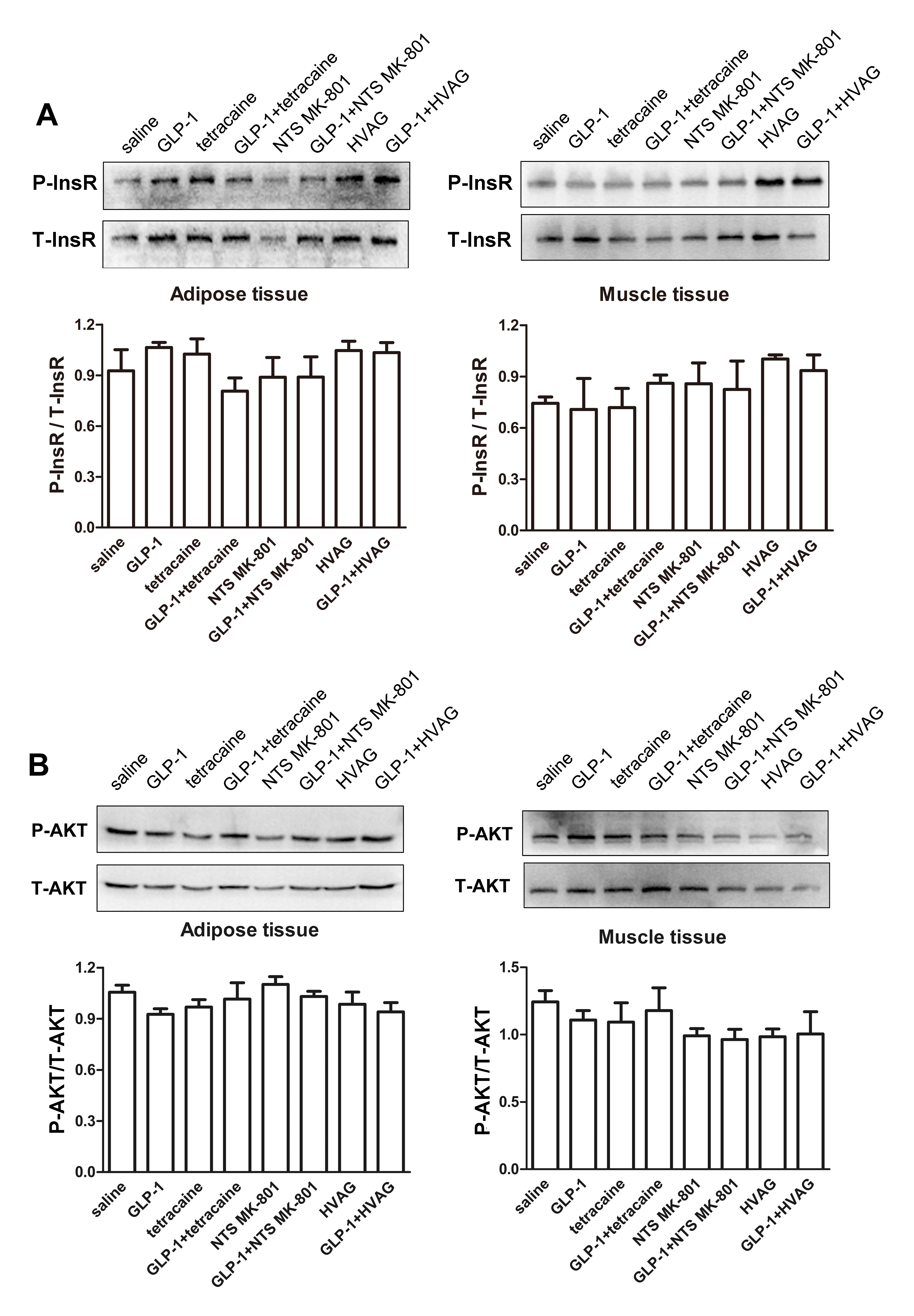
**

**Figure S5. Duodenal GLP-1 infusion fails to augment insulin signaling in adipose and muscle tissues. (A)** Representative western blots and ratios of phosphorylated InsR to total InsR in adipose and muscle tissues from experiments shown in Fig. 2B. **(B)** Representative western blots and ratios of phosphorylated AKT to total AKT in adipose and muscle tissues from experiments shown in Fig. 2B. Data are means ± SEM.

**
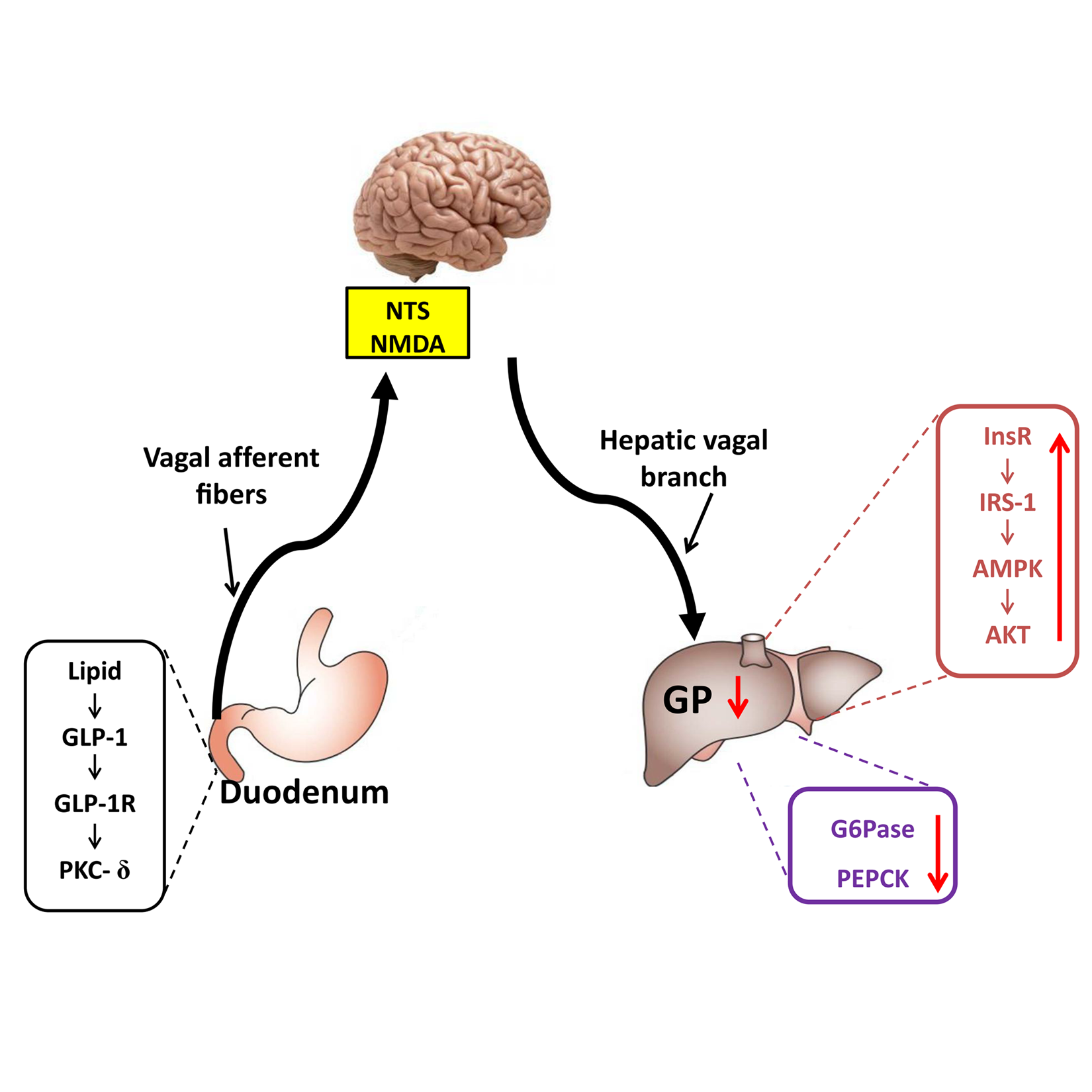
**

**Figure S6. A schematic model showing the effects of duodenal GLP-1 on hepatic glucose fluxes.** The upper intestine nutrient, such as lipid, stimulates the release of GLP-1 from the mucosal cells by paracrineaction. GLP-1 binds to GLP-1 receptors on the vagal afferents innervating the duodenum and activates mucosal PKC-δ. This signaling is delivered to the NTS by vagus afferent nerve, and activates the neurons in this hindbrain region and NMDA receptors. Finally, the signaling is relayed from the NTS to the liver via the efferent branch of the vagal nerve to lower HGP.


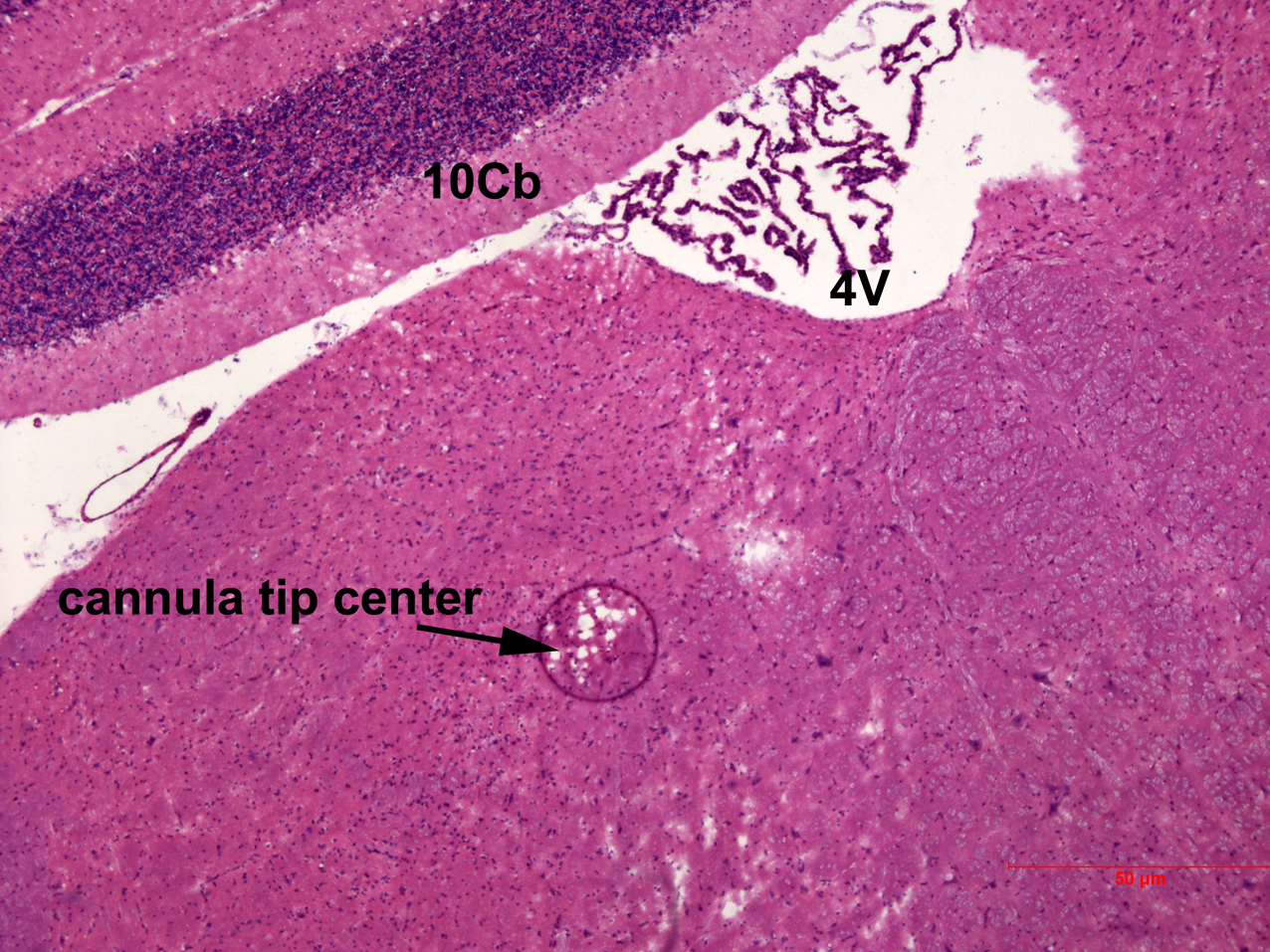


**Figure S7.** Photograph of a representative histologic section demonstrating a cannula tip centered in the left medial subnucleus of the NTS. Abbreviations: 10Cb, 10th cerebellar lobule; 4V, fourth ventricle. Original magnification, ×40.

**Supplementary Tables**

**Table S1. Plasma metabolic parameters under basal and clamped conditions**

|  | Insulin (ng/ml) | Glucose (mmol/l) | TG  (mmol/l) | TC  (mmol/l) | FFA (mmol/L) |
| --- | --- | --- | --- | --- | --- |
| **saline** | | |  |  |  |
| Basal | 0.83 ± 0.14 | 7.0 ± 0.4 | 0.71 ± 0.09 | 1.79 ± 0.15 | 1.07 ± 0.17 |
| Clamp | 0.80 ± 0.12 | 7.5 ± 0.3 | 0.68 ± 0.08 | 1.66 ± 0.14 | 1.03 ± 0.21 |
|  | | |  |  |  |
| **GLP-1** | | |  |  |  |
| Basal | 0.81 ± 0.28 | 7.1 ± 0.2 | 0.65 ± 0.10 | 1.72 ± 0.15 | 0.98 ± 0.03 |
| Clamp | 0.88 ± 0.21 | 6.9 ± 0.3 | 0.51 ± 0.08 | 1.60 ± 0.17 | 0.99 ± 0.18 |
|  | | |  |  |  |
| **Ex-9** | | |  |  |  |
| Basal | 0.80 ± 0.23 | 7.2 ± 0.1 | 0.51 ± 0.07 | 1.67 ± 0.15 | 1.04 ± 0.21 |
| Clamp | 0.83 ± 0.25 | 6.9 ± 0.5 | 0.70 ± 0.11 | 1.74 ± 0.12 | 1.08 ± 0.06 |
|  | | |  |  |  |
| **GLP-1+Ex-9** | | |  |  |  |
| Basal | 0.82 ± 0.13 | 7.1 ± 0.6 | 0.57 ± 0.07 | 1.72 ± 0.08 | 1.03 ± 0.08 |
| Clamp | 0.86 ± 0.27 | 6.6 ± 0.5 | 0.65 ± 0.06 | 1.58 ± 0.08 | 0.87 ± 0.17 |
|  | | |  |  |  |
| **Tetracaine** | | |  |  |  |
| Basal | 0.87 ± 0.23 | 6.5 ± 0.2 | 0.73 ± 0.07 | 1.58 ± 0.18 | 1.11 ± 0.16 |
| Clamp | 0.89 ± 0.19 | 6.2 ± 0.2 | 0.70 ± 0.06 | 1.65 ± 0.16 | 1.02 ± 0.18 |
|  | | |  |  |  |
| **GLP-1+ Tetracaine** | | |  |  |  |
| Basal | 0.89 ± 0.12 | 7.5 ± 0.4 | 0.64 ± 0.10 | 1.87 ± 0.07 | 1.14 ± 0.15 |
| Clamp | 0.87 ± 0.29 | 7.2 ± 0.5 | 0.59 ± 0.08 | 1.80 ± 0.08 | 1.09 ± 0.14 |
|  | | |  |  |  |
| **NTS MK-801** | | |  |  |  |
| Basal | 1.01 ± 0.27 | 7.8 ± 0.4 | 0.56 ± 0.13 | 1.51 ± 0.07 | 1.09 ± 0.18 |
| Clamp | 0.82 ± 0.31 | 7.6 ± 0.4 | 0.58 ± 0.18 | 1.57 ± 0.14 | 0.82 ± 0.28 |
|  | | |  |  |  |
| **GLP-1+ NTS MK-801** | | |  |  |  |
| Basal | 0.91 ± 0.21 | 6.7 ± 0.6 | 0.51 ± 0.08 | 1.14 ± 0.11 | 1.03 ± 0.10 |
| Clamp | 0.82 ± 0.22 | 6.5 ± 0.7 | 0.57 ± 0.09 | 1.21 ± 0.13 | 0.83 ± 0.06 |
|  |  | |  |  |  |
| **HVAG** | | |  |  |  |
| Basal | 0.91 ± 0.24 | 7.8 ± 0.6 | 0.55 ± 0.06 | 1.58 ± 0.17 | 1.04 ± 0.10 |
| Clamp | 0.94 ± 0.14 | 7.6 ± 0.5 | 0.56 ± 0.07 | 1.65 ± 0.20 | 1.17 ± 0.05 |
|  | | |  |  |  |
| **GLP-1+ HVAG** | | |  |  |  |
| Basal | 0.99 ± 0.27 | 8.1 ± 0.4 | 0.53 ± 0.09 | 1.59 ± 0.17 | 1.16 ± 0.07 |
| Clamp | 1.08 ± 0.13 | 7.7 ± 0.8 | 0.50 ± 0.07 | 1.65 ± 0.20 | 0.98 ± 0.07 |
|  | | |  |  |  |
| **Lipid** | | |  |  |  |
| Basal | 0.90 ± 0.13 | 7.0 ± 0.3 | 0.51 ± 0.08 | 1.84 ± 0.15 | 1.10 ± 0.10 |
| Clamp | 0.93 ± 0.10 | 7.6 ± 0.3 | 0.51 ± 0.10 | 1.87 ± 0.08 | 1.13 ± 0.19 |
|  | | |  |  |  |
| **Lipid+ Ex-9** | | |  |  |  |
| Basal | 0.79 ± 0.19 | 7.3 ± 0.3 | 0.59 ± 0.17 | 1.55 ± 0.14 | 0.92 ± 0.12 |
| Clamp | 0.85 ± 0.22 | 7.2 ± 0.2 | 0.53 ± 0.15 | 1.45 ± 0.29 | 1.01 ± 0.15 |
|  | | |  |  |  |
| **Rottlerin** | | |  |  |  |
| Basal | 0.92 ± 0.15 | 6.9 ± 0.5 | 0.57 ± 0.07 | 1.43 ± 0.11 | 1.15 ± 0.09 |
| Clamp | 0.90 ± 0.15 | 6.5 ± 0.6 | 0.53 ± 0.11 | 1.36 ± 0.12 | 0.99 ± 0.17 |
|  | | |  |  |  |
| **Rottlerin+ GLP-1** | | |  |  |  |
| Basal | 0.91 ± 0.21 | 7.2 ± 0.4 | 0.61 ± 0.22 | 1.77 ± 0.21 | 1.09 ± 0.21 |
| Clamp | 0.86 ± 0.17 | 7.9 ± 0.3 | 0.68 ± 0.15 | 1.64 ± 0.16 | 0.98 ± 0.10 |
|  | | |  |  |  |
| **Exenatide** | | |  |  |  |
| Basal | 0.91±0.16 | 7.9 ± 0.4 | 0.50 ± 0.07 | 1.88 ± 0.18 | 0.98 ± 0.15 |
| Clamp | 0.98±0.14 | 7.7 ± 0.4 | 0.53 ± 0.03 | 1.85 ± 0.22 | 0.94 ± 0.13 |

GLP-1, Liraglutide; Ex-9, **Exendin Fragment 9-39;** TG, Triglyceride; TC, Total cholesterol; FFA, free fatty acid; NTS, Nucleus of the solitary tract. Data are means ± SEM.

**Table S2 . Circulating and portal vein GLP-1 concentrations (μg/L)**

|  | Saline | Liraglutide |
| --- | --- | --- |
| Circulation | 6.4 ± 1.0 | 6.20 ± 1.3 |
| Portal vein | 9.3 ± 0.3 | 11.7 ±1.4 |

Data are means ± SEM.
